# Environment spectrum and coherence behaviours in a rare-earth doped crystal for quantum memory

**DOI:** 10.1038/s41598-017-18229-6

**Published:** 2017-12-21

**Authors:** Bo Gong, Tao Tu, Zhong-Quan Zhou, Xing-Yu Zhu, Chuan-Feng Li, Guang-Can Guo

**Affiliations:** 1 Key Lab of Quantum Information, Chinese Academy of Sciences, University of Science and Technology of China, Hefei, 230026 China; 2 Department of Physics and Astronomy, University of California at Los Angeles, California, 90095 USA

## Abstract

We theoretically investigate the dynamics of environment and coherence behaviours of the central ion in a quantum memory based on a rare-earth doped crystal. The interactions between the central ion and the bath spins suppress the flip-flop rate of the neighbour bath spins and yield a specific environment spectral density S(*ω*). Under dynamical decoupling pulses, this spectrum provides a general scaling for the coherence envelope and coherence time, which significantly extend over a range on an hour-long time scale. The characterized environment spectrum with ultra-long coherence time can be used to implement various quantum communication and information processing protocols.

## Introduction

Quantum memories have been proposed as an essential component for quantum communication networks and quantum information processing^1^, where they are used as interfaces between flying and stationary qubits. Experiments with multiple physical platforms are currently in progress to realize prototypes of optical quantum memories^2,3^. Theoretically, there are several performance criteria/figures of merit (e.g., storage time, efficiency, fidelity and capacity) for such quantum systems^4^. The key requirement is the ability to store quantum states for times that are long compared to the direct transmission time of the channel.

Rare-earth doped crystals are particularly attractive for quantum memory applications with high fidelity mapping of optical quantum states onto the ions in the crystal^5–7^, and efficient storage of single photons and quantum entanglement^8–14^. The ability to store the optical states in long-lived collective spin excitations in solids is essential for many applications^15–18^). This spin-wave storage scheme also gives access to much longer storage times via further dynamical decoupling manipulations. Very recently, the effectiveness of dynamical decoupling sequences in mitigating the decoherence of hyperfine spin states of the europium-ion dopant in yttrium orthosilicate (Eu^3^ + : Y_2_SiO_5_) was demonstrated^19^. However, much less is known about the characterization of the spectral density of the environment *S*(*ω*). The knowledge of *S*(*ω*) has a broad range of applications, enabling the design of the optimal coherent-control method^20–24^, providing a wealth of information to unravel the underlying many-body physics^25^, and directly improving the storage time of quantum memory.

**Figure 1:**
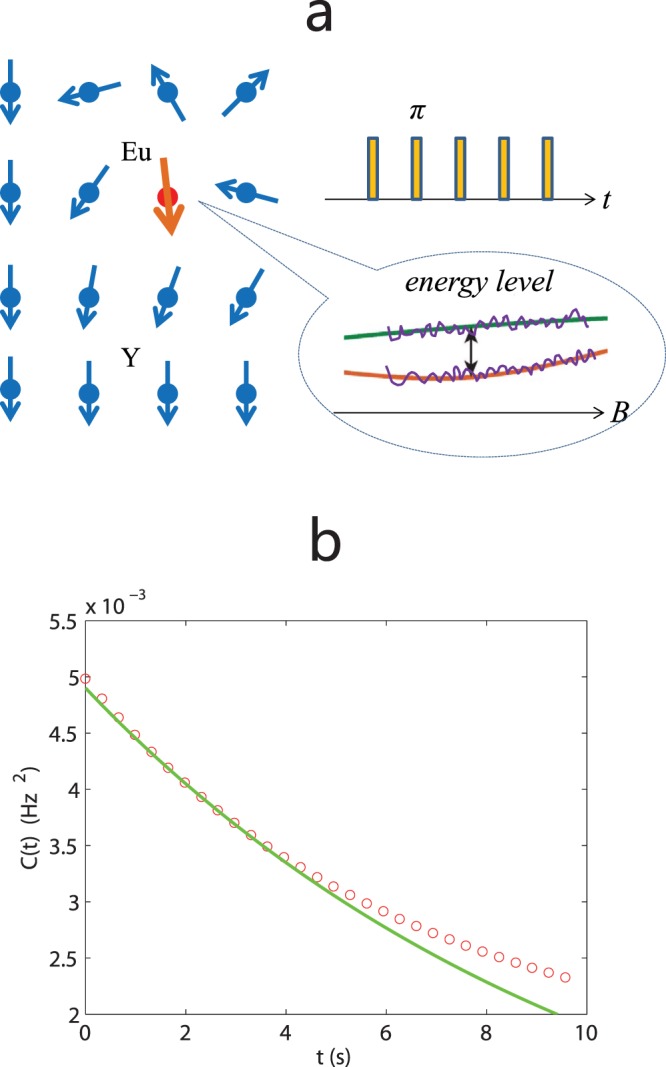
(**a**) Illustration of the coherent spectroscopic method to probe the environment dynamics of Y bath spins around the Eu ion in a typical quantum memory. Dynamical decoupling *π*-pulse sequences are applied to probe the coherence of the Eu ion. The environment effects are described as fluctuations about the energy levels of the Eu ion. (**b**) Numerically calculated environment correlation function *C*(*t*) (red circles) and the fitted shape ${b}^{2}\exp (-t/{\tau }_{c})$ (green solid line) with *b* = 0.07 Hz and long correlation time *τ*_*c*_ = 12 s.

In this article, we theoretically investigate the environment spectra and the corresponding coherence behaviours in a typical quantum memory with hyperfine structures of Eu ions occupying yttrium positions in Y_2_SiO_5_ [Fig. 1(a)]. First, we model the composite system of the Eu ion and Y bath to obtain a Lorentzian-shape spectral density *S*(*ω*) with significantly long correlation time *τ*_*c*_. This specific environment spectrum is a result of the interplay between the central Eu ion and Y bath spins, which suppresses the bath dynamics. Then, we theoretically characterize the environment spectrum using the filter properties of dynamical decoupling sequences. The approach provides the scaling behaviours of the coherence envelope and coherence time depending on the environment spectral density *S*(*ω*). In particular, we theoretically show that the combined effect of this slow-bath dynamics (i.e., large value of *τ*_*c*_) and dynamical decoupling techniques enables the scaling behaviours to span over a wide range on an ultra-long-term scale (an hour-long time scale, which is orders of magnitude longer than that of any other systems suitable for quantum memory). These results open the door for long-lived storage and represent an important step in using solid state quantum memories in scalable quantum information processes.

## Results

### Modelling the environment dynamics and its spectrum

The investigated quantum memory based on the rare-earth-doped crystal system is defined by the hyperfine energy levels of the Eu^3+^ ions occupying yttrium positions in Y_2_SiO_5_, as shown in Fig. 1(a). Realistic experiments are commonly performed in a refrigerator at liquid helium temperatures. The doubly degenerate hyperfine states ($|\pm 3/2\rangle$) are split by the applied magnetic field. The coherence properties of the $|+3/2\rangle \leftrightarrow |-3/2\rangle$ transition are probed by preparing a population difference between the $|+3/2\rangle$ and $|-3/2\rangle$ states, then employing dynamical decoupling pulses and detecting the signal [Fig. 1(a)]. The dynamical decoupling sequences of interest are manipulated at the radio frequency (RF) frequency, and the resultant ion coherence is determined by Raman heterodyne detection using an additional optical pulse at the rephasing time.

To understand this system, we focus on a dilute dipolar-coupled model where a central ion (e.g., an impurity Eu ion) is coupled to an environment of many spins (e.g., Y nuclear spins in a Y_2_SiO_5_ crystal)^25^. This situation encompasses a wide range of interesting solid-state quantum memories using rare-earth-doped crystals^26^. The total Hamiltonian of the central ion and bath spins is:

$${H}_{t}={H}_{s}+{H}_{e},$$

$${H}_{s}={{\gamma }}_{s}B{S}_{z}+\sum _{i}{b}_{i}{I}_{i}^{z}{S}_{z},$$

1 $${H}_{e}={\gamma }_{I}B\sum _{i}{I}_{i}^{z}+\sum _{i < ,\,j}{d}_{ij}\mathrm{(4}{I}_{i}^{z}{I}_{j}^{z}-{I}_{i}^{+}{I}_{j}^{-}-{I}_{i}^{-}{I}_{j}^{+})$$

where *S* and *I*_*i*_ are the central ion and bath spin operators, *b*_*i*_ = $\frac{1}{4\pi }{\gamma }_{s}{\gamma }_{I}\hslash \frac{1-3{\cos }^{2}{\theta }_{i}}{{r}_{i}^{3}}$ is the coupling constant and ${d}_{ij}=\frac{1}{4\pi }{\gamma }_{I}^{2}\hslash \frac{1-3{\cos }^{2}{\theta }_{ij}}{{r}_{ij}^{3}}$ is the intra-bath interaction. Here *γ*_*s*_ and *γ*_*I*_ are the gyromagnetic ratios; *r*_*ij*_ and *r*_*i*_ are the distances between two spins involved; *θ*_*ij*_ and *θ*_*i*_ are the angles between the vector that connects the two spins and the magnetic field.

Next, we performed realistic calculations of Hamiltonian Eq. (1) to identify the environment dynamics and its effect on the central ion. The Y bath dynamics at the Eu ion site creates a fluctuating Zeeman field $\delta B={\sum }_{i}{b}_{i}{I}_{i}^{z}$, which drives the decoherence transition between the $|+3/2\rangle$ and $|-3/2\rangle$ states of the Eu ion. The time-dependent correlation function for the bath spins is given by

2 $$\begin{array}{ccl}\langle \delta B(t)\delta B\mathrm{(0)}\rangle  & = & \langle \sum _{i}{b}_{i}{I}_{i}^{z}(t)\sum _{j}{b}_{j}{I}_{j}^{z}(t)\rangle \\  & = & \sum _{i}{b}_{i}^{2}\langle {I}_{i}^{z}(t){I}_{i}^{z}\mathrm{(0)}\rangle +\sum _{i,j\ne i}{b}_{i}{b}_{j}\langle {I}_{i}^{z}(t){I}_{j}^{z}\mathrm{(0)}\rangle .\end{array}$$

The bath-spin effect or operator ${I}_{i}^{z}(t)$ in the Heisenberg representation is determined by the Hamiltonian Eq. (1). Since *H*_*e*_ causes the flip-flop transitions inside the bath, we use a “pair-approximation” as follows^25,27^:

$$\langle {I}_{i}^{z}(t){I}_{i}^{z}\mathrm{(0)}\rangle \approx \sum _{j\ne i}{\langle {I}_{i}^{z}(t){I}_{i}^{z}\mathrm{(0)}\rangle }_{(ij)},$$

3 $$\langle {I}_{i}^{z}(t){I}_{j}^{z}\mathrm{(0)}\rangle \approx {\langle {I}_{i}^{z}(t){I}_{j}^{z}\mathrm{(0)}\rangle }_{(ij)}$$

where 〈..〉_(*ij*)_ denotes an average restricted to the (*ij*) pair space. In the pair-correlation approximation, it is assumed that each flip-flop process is independent of all other processes, i.e., the Hilbert-space is substituted by independent pair spaces (*ij*) comprising any two bath spin combinations. We note that the present approximation corresponds to the lowest order of exact approaches such as cluster expansion methods^28,29^.

Now, the time evolution of the operator can be expressed as

4 $$\langle \delta B(t)\delta B\mathrm{(0)}\rangle \approx \sum _{i < j}{\langle \delta {B}_{ij}(t)\delta {B}_{ij}\mathrm{(0)}\rangle }_{(ij)}$$

where $\delta {B}_{ij}={b}_{i}{I}_{i}^{z}+{b}_{j}{I}_{j}^{z}$ can be analytically evaluated in the subspace denoted by (*ij*). The rate for a flip-flop process between two Y bath spins is

5 $${R}_{ij}={T}_{ij}^{-1}=\frac{\sqrt{2\pi }}{4}\frac{{d}_{ij}^{2}}{{\sigma }_{ij}}\exp (\,-\,\frac{{{\rm{\Delta }}}_{ij}^{2}}{2{\sigma }_{ij}^{2}}),$$

where $${{\rm{\Delta }}}_{ij}=\frac{1}{2}({b}_{i}-{b}_{j})$$ is the frequency shift felt by the Eu ion when one flip-flop event occurs, and $${\sigma }_{ij}^{2}=$$$$4{\sum }_{k\ne i,j}{({d}_{ik}-{d}_{jk})}^{2}$$ is the linewidth for the flip-flop. Therefore, we obtain a correlation function of bath spins

6 $$\langle \delta B(t)\delta B\mathrm{(0)}\rangle =\frac{1}{4}\sum _{i}{b}_{i}^{2}\sum _{i < j}{P}_{ij}\exp (\,-\,\frac{2t}{{T}_{ij}}),$$

where ${P}_{ij}={R}_{ij}{{\rm{\Delta }}}_{ij}^{2}/{\sum }_{n < m}{R}_{nm}{{\rm{\Delta }}}_{nm}^{2}$ (for explicit formula derivations, we refer the reader to the Supplementary information).

We evaluate the environment effects as noise terms in the Hamiltonian of the ion^20^,

7 $${H}_{eff}={\varepsilon }_{0}{S}_{z}+{\varepsilon }_{1}\delta B{S}_{z}+{\varepsilon }_{2}{(\delta B)}^{2}{S}_{z}+\mathrm{..}.$$

where we expand the Hamiltonian in the perturbation of the *δB*: ${\varepsilon }_{1}=\frac{\partial {\varepsilon }_{0}}{\partial B}$ and ${\varepsilon }_{2}=\frac{1}{2}\frac{{\partial }^{2}{\varepsilon }_{0}}{\partial {B}^{2}}$. The experiments are usually performed around the region where the first-order term of the transition frequency with respect to the magnetic field is very small, i.e., the so-called ZEFOZ (zero first-order Zeeman transition) region^19^. We conclude that the spin bath can be treated as a noise field *δε* with an environment spectral density

8 $$S(\omega )={\int }_{-\infty }^{\infty }dt\langle \delta \varepsilon (t)\delta \varepsilon \mathrm{(0)}\rangle {e}^{i\omega t}$$

which acts on the central ion.

### Characterization of the specific long correlation time

The environment noise correlation function *C*(*t*) = 〈*δε*(*t*)*δε*(0)〉 is numerically calculated by placing the central ion and bath spins in a crystal lattice and using the realistic input parameters *γ*_*s*_ and *γ*_*I*_ (the details of the simulation method are provided in the Supplementary information). As shown in Fig. 1(b), the calculated environment correlation is fit to a shape ${b}^{2}\exp (\,-\,t/{\tau }_{c})$ with amplitude *b* = 0.07 Hz and correlation time *τ*_*c*_ = 12 s. Therefore, the resulting spectral density of the Y bath spins can be assumed to be Lorentzian: $S(\omega )=\frac{2{b}^{2}{\tau }_{c}}{{\omega }^{2}{\tau }_{c}^{2}+1}$. This environment spectrum is characterized by two parameters: *b* is the average coupling strength of the Y bath to the Eu ion, and *τ*_*c*_ is the correlation time of the Y bath spins, which is related to their flip-flop rate. We also note that the simulated correlation function will deviate from the form ${b}^{2}\exp (\,-\,t/{\tau }_{c})$ in the long term, indicating the small discrepancy between the realistic system and Lorentzian model.

In general, there are several characteristic time quantities in this composite system of the central ion and bath spins. On the one hand, *τ*_*c*_ is the correlation time of the bath, which describes the flip-flop process of the Y bath spins. On the other hand, *τ*_*d*_ and *τ*_*b*_ are given by the inverses of the intra-bath interaction *d*_*ij*_ and central ion-bath coupling *b*_*i*_, respectively. Because of the energy scales *d*_*ij*_ ~ 10 Hz and *b*_*i*_ ~ 2 kHz (the Eu ion with the nearest neighbour Y spins), we can directly infer that *τ*_*d*_ ~ 0.1 s and *τ*_*b*_ ~ 0.5 ms. Surprisingly, the correlation time *τ*_*c*_ ~ 12 s is several orders of magnitude longer than any other characteristic time of the present system, which is attributed to the so-called frozen core mechanism^19^: the significantly large interaction between the central Eu ion and the Y bath spins makes the Larmor frequencies of neighbouring Y spins detuned from one another. This specific central ion-bath interaction suppresses the flip-flop dynamics of the Y bath spins, which is explicitly given by the exponential reduced factor $\exp (-\frac{{{\rm{\Delta }}}_{ij}^{2}}{2{\sigma }_{ij}^{2}})$ in Eq. (5).

### Scaling behaviours for the ultra-long coherence time

The coherence lost due to evolution under environment noise *δε* can be partially restored using a Hahn echo by pulsing at time *t*/2, where the application of an instantaneous RF pulse on the ion drives a *π*-rotation, which changes the sign of the acquired phase. Returning to evolution under *δε* for an equal time *t*/2 cancels the phase acquired because of the low-frequency (*ω* < 2/*t*) end of the spectrum of fluctuations of *δε*. Dynamical decoupling using a series of *π*-pulses enables the efficient removal of the low-frequency end of the environment noise spectrum. For example, the CPMG sequence uses evenly spaced *π*-pulses with a half interval before the first and after the last *π*-pulses.

**Figure 2:**
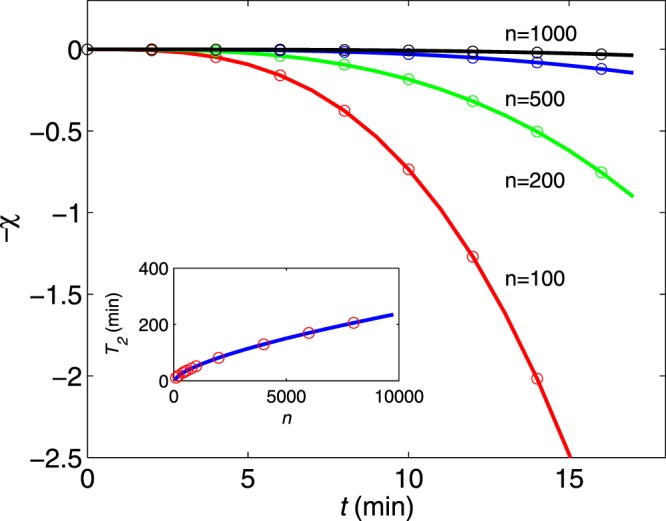
Numerical simulations (circles) of the coherence envelope as a function of time for the CPMG sequences with pulse numbers *n* = 100, 200, 500, and 1000. The solid lines are the calculated values using the analytic formula (*t*/*T*_2_)^*α*^ with *α* = 3. Note that the scaling is extended over very long time scales. Insert: Extracted coherence time *T*_2_ for the CPMG pulse number. The solid line is also obtained by the analytic formula *T*_2_ ~ (*n*)^*γ*^ with *γ* = 2/3.

We study the environment noise affecting the energy splitting of the ion states, which makes the coherence (off-diagonal elements of the density matrix) decay as $\exp [-\chi (t)]$ (circles in Fig. 2). Here, we fix the number of CPMG pulses sequences at *n* = 100, 200, 500, and 1000 and vary the pulse interval *τ*. The evolution of the central ion density matrix elements is obtained from a numerical simulation of the time-dependent Schrodinger equation. The value of the effective noise *δε*(*t*) at each time step is randomly sampled using the previous value and transition probability for the environment process with correlation function *C*(*t*). The central ion density matrix elements are averaged over 500 realizations of the *δε*(*t*) process (the details of the simulation method are provided in the Supplementary information).

The dynamical decoupling scheme applies a sequence of *π*-pulses as a high-pass filter. Thus, the spectral information *S*(*ω*) about the environment noise can be presented in the coherece behaviours^20,30–33^. To understand these simulation results, we consider^20^

9 $$\chi (t)={\int }_{0}^{\infty }\frac{{\rm{d}}\omega }{\pi }S(\omega )\frac{F(\omega t)}{{\omega }^{2}}$$

where *F*(*ωt*) is the filter function determined by the sequence of *π*-pulses driving the system. For a Lorentzian environment spectral density $S(\omega )=\frac{2{b}^{2}{\tau }_{c}}{{\omega }^{2}{\tau }_{c}^{2}+1}$, in the case of a short correlation time (*τ*_*c*_ ≪ *τ*), the environment noise does not show any memory effects on the relevant time scale. Therefore, the dynamical decoupling sequence is inefficient, and the coherence does not improve with the number of pulses.

In the opposite case of a long correlation time (*τ*_*c*_ ≫ *τ*), in the large-*n* limit, Eq. (9) gives

10 $$\chi (t)\approx \frac{{(b{\tau }_{c})}^{2}}{12{n}^{2}}{(\frac{t}{{\tau }_{c}})}^{3}$$

(the analytic expressions for the filter function are provided in the Supplementary information). This formula originates from the feature of the CPMG filtering of the Lorentzian spectrum, which suppresses the low-*ω* contributions to the integral in Eq. (9), and only the tail part (*ω* > 1/*τ*_*c*_) of the Lorentzian spectral density *S*(*ω*) contributes to the decoherence *χ*(*t*). Then, most of the decoherence occurs as an *χ* ~ *t*^3^ term. In particular, we can re-express it as

11 $$\chi (t)\approx {(\frac{t}{{T}_{2}})}^{3},{T}_{2}={(\frac{12{\tau }_{c}}{{b}^{2}})}^{\frac{1}{3}}{n}^{\frac{2}{3}}$$

Thus, the scaling behaviours apply, namely, *χ*(*t*) ~ (*t*/*T*_2_)^*α*^ and *T*_2_ ~ (*n*)^*γ*^ with *α* = 3 and *γ* = 2/3. Assuming this form of *S*(*ω*) with *b* = 0.07 Hz and *τ*_*c*_ = 12 s and using the analytic formula Eq. (11), we calculate the *χ* values for CPMG pulse sequences (solid lines in Fig. 2).

The diagrams show two remarkable features. First, the scaling behaviours notably accurately describe the simulation results regardless of the dynamical decoupling pulse details. Thus, *S*(*ω*) is related to *α* and *γ*, and these scaling behaviours provide a wealth of information about the spectrum of the environment. Second, the scaling behaviours are significantly robust over a range of coherence time spanning an hour-long time scale. This robust scaling is a result of the combined effect of slow-bath dynamics and dynamical decoupling sequences: the specific large bath correlation time *τ*_*c*_ and long pulse sequences *n* significantly increase the coherence time *T*_2_ from 1 minute to 200 minutes, as shown in Fig. 2. The maximal value of *T*_2_ (200 min) is three orders of magnitude longer than *τ*_*c*_ (12 s).

### Universal scaling for various cases

**Figure 3:**
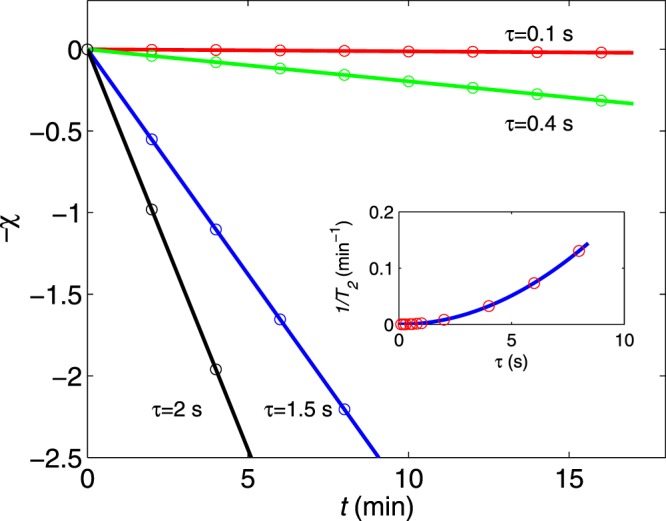
Simulation data (circles) of the coherence envelope as a function of time for the CPMG sequences with pulse intervals of *τ* = 0.1 s, 0.4 s, 1.2 s, and 2 s. The solid lines are obtained using the scaling expression (*t*/*T*_2_)^*β*^ with *β* = 1. Insert: Extracted 1/*T*_2_ for the CPMG pulse interval. The solid line is also the scaling function 1/*T*_2_ ~ *τ*^*δ*^ with *δ* = 2.

For applications in quantum information processing, the scaling must be universal, i.e., it can be preserved for other protocols of interest. As shown in Fig. 3, the coherence *χ*(*t*) is simulated for a number of CPMG pulses with pulse intervals of *τ* = 0.1 s, 0.4 s, 1.2 s, and 2 s. Using the outlined analytical derivations, we find that in the case of fixed *τ* and varied *n*, the scaling behaviours of coherence hold,

12 $$\chi (t)\approx {(\frac{t}{{T}_{2}})}^{\beta },\frac{1}{{T}_{2}}\sim {\tau }^{\delta }$$

where *β* = 1 and *δ* = 2. We also note that the analytically calculated decoherence rates are consistent with the experimentally measured value^19^. For example, the calculated coherence time *T*_2_ for *τ* = 10 s is 294 s, which is consistent with the measured coherence time of the CPMG decay curve (240 s).

**Figure 4:**
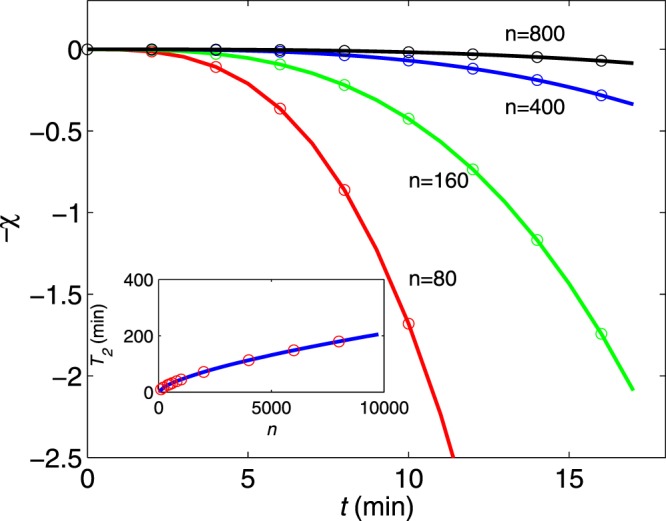
Numerical simulations (circles) of the coherence envelope *χ* (main diagram) and corresponding coherence time *T*_2_ (insert) for UDD sequences. The solid lines are fits to the scaling behaviours of *χ* ~ (*t*/*T*_2_)^*α*^ with *α* = 1 and *T*_2_ ~ *n*^*γ*^ with *γ* = 2/3.

Further support is found by calculating the expected dependence of *χ*(*t*) for other pulse sequences such as the UDD^34^. Since there is no analytic formula, we can use the direct numerical simulations and fit the coherence envelopes to the scaling form. As shown in Fig. 4, we obtain similar scaling behaviours as in the case of the CPMG sequence. This result indicates that the scaling behaviours for the specific environment spectral density *S*(*ω*) apply in a wide range of dynamical decoupling schemes. Although our theoretical analysis was performed for a specific system (a europium-doped crystal), the method is independent of the physical encoding of the quantum information and has the potential for wider applications (a similar analysis for a praseodymium-doped crystal is performed in the Supplementary information).

## Discussion

In summary, we have modelled the crucial features of the environment spectrum and corresponding coherence behaviours in a quantum memory based on rare-earth-doped crystals. We have revealed the significant suppression of the bath spin dynamics, which can be explained by the interactions between the central Eu ion and proximal Y bath spins. This intrinsic slow-bath dynamics (i.e., long correlation time) and implementation of the dynamical decoupling sequences enables the caling expressions of the coherence envelope and coherence time, which span over a range on the ultra-long time scale. These theoretical results can be directly applied to explain the underlying mechanism for the observed hour-long coherence time in a recent experiment^19^. Furthermore, rare-earth crystals have recently demonstrated a series of highlighted progress in terms of long-lived spin states^15–18^, large efficiencies^9^, high fidelity^5–7^, and multimode capacity^10,14^, for quantum storage. However, the full capabilities of all figures of merit for a solid-state quantum memory have not been exploited, and challenges remains. For example, using the promising properties of the environment spectrum enables one to design a quantum memory with simultaneously high efficiency and long storage time.

## Electronic supplementary material

Supplementary Information
