# Publisher Correction: Environment spectrum and coherence behaviours in a rare-earth doped crystal for quantum memory

**DOI:** 10.1038/s41598-018-35597-9

**Published:** 2018-11-14

**Authors:** Bo Gong, Tao Tu, Zhong-Quan Zhou, Xing-Yu Zhu, Chuan-Feng Li, Guang-Can Guo

**Affiliations:** 1 Key Lab of Quantum Information, Chinese Academy of Sciences, University of Science and Technology of China, Hefei, 230026 China; 2 Department of Physics and Astronomy, University of California at Los Angeles, California, 90095 USA

Correction to: *Scientific Reports* 10.1038/s41598-017-18229-6, published online 21 December 2017

In the original version of this Article, Guang-Can Guo, and not Chuan-Feng Li, was incorrectly listed as a corresponding author. The correct corresponding authors for this Article are Tao Tu and Chuang Feng Li. Correspondence and requests for materials should be addressed to Tao Tu (tutao@ustc.edu.cn) or Chuan-Feng Li (cfli@ustc.edu.cn). This error has now been corrected in the HTML and PDF versions of the Article.

