# Supplementary material for: Environment spectrum and coherence behaviours in a rare-earth doped crystal for quantum memory
Source: Sci Rep. 2017 Dec 21;7:18030. doi: 10.1038/s41598-017-18229-6 (PMC5740147; doi:10.1038/s41598-017-18229-6)
Supplement: Supplementary file 1 — Supplementary Information [file 41598_2017_18229_MOESM1_ESM.pdf]

Environment spectrum and coherence behaviours in a rare-earth doped crystal for quantum memory : Supplemental information

B. Gong, T. Tu, Z. Q. Zhou, X. Y. Zhu, C. F. Li, and G. C. Guo

(Dated: November 10, 2017)

REALISTIC CALCULATIONS OF THE ENVIRONMENT NOISE SPECTRUM

In the present paper we focus on quantum memories for light using the hyperfine states of rare-earth optical centres in crystals. Such a coupled system of the central ion and the bath nuclear spins can be described by [1]

$$\begin{aligned} H_t &= H_s + H_i + H_e, \\ H_s &= \gamma_s B S_z, \\ H_i &= \textstyle\sum_i b_i I_i^z S_z, \text{ with } b_i = \frac{\mu_0}{4\pi}\frac{\gamma_I \gamma_s \hbar}{r_i^3}(1 - 3\cos^2\theta_i) \\ H_e &= \gamma_I B \textstyle\sum_i I_i^z + \sum_{i<,j} d_{ij}(4I_i^z I_j^z - I_i^+ I_j^- - I_i^- I_j^+), \text{ with } d_{ij} = \frac{\mu_0}{4\pi}\frac{\gamma_I^2 \hbar}{r_{ij}^3}(1 - 3\cos^2\theta_{ij}) \end{aligned} \tag{S1}$$

Here H_s describes the central ion Hamiltonian, H_i includes the dipolar coupling of the environment spins to the central ion, and H_e represents the magnetic dipolar interaction between environment nuclear spins. S denotes the operator of the central ion, I_i the operators of the environment nuclear spins, r_{ij} the distance between spin i and j, and r_i the one between environment spin i and the central ion, θ_{ij} and θ_i the angles between the vector connecting the two coupled spins involved and the external magnetic field.

From Eq. (S1) the operator for the effective field on the central ion follow as

$$\delta B = \sum_i b_i I_i^z, \tag{S2}$$

which allows for calculation of the environment noise spectrum $S(\omega)$ defined as

$$S(\omega) = \int_{-\infty}^{\infty} dt \, \langle \delta B(t) \delta B(0) \rangle \, e^{i\omega t}. \tag{S3}$$

Various approaches, in particular the phenomenological assumption of Ornstein-Uhlenbeck process has been developed for calculating the environment noise spectrum and the corresponding parameters [2, 3]. Here we use the method [1, 4, 5] which systematically deals with the collective quantum evolution of the dipolar coupled environment spins, allows realistic calculations of environment spectrum properties without using any phenomenological assumption.

The environment spin noise is given by the time-dependent correlation function as

$$\begin{aligned} \langle \delta B(t) \delta B(0) \rangle &= \left\langle \textstyle\sum_i b_i I_i^z(t) \sum_j b_j I_j^z(t) \right\rangle \\ &= \textstyle\sum_i b_i^2 \left\langle I_i^z(t) I_i^z(0) \right\rangle + \sum_{i,j\neq i} b_i b_j \left\langle I_i^z(t) I_j^z(0) \right\rangle . \end{aligned} \tag{S4}$$

and is in turn determined by the effective Hamiltonian H_e. When any I_i flips, a corresponding I_j must flop in the opposite direction. Thus these flip-flop events construct the collective quantum evolution of the dipolar coupled environment spins, and a reasonable description is introduced by

$$\begin{aligned} \left\langle I_i^z(t) I_i^z(0) \right\rangle &\approx \textstyle\sum_{j\neq i} \left\langle I_i^z(t) I_i^z(0) \right\rangle_{(ij)}, \\ \left\langle I_i^z(t) I_j^z(0) \right\rangle &\approx \left\langle I_i^z(t) I_j^z(0) \right\rangle_{(ij)}, \end{aligned} \tag{S5}$$

i.e. ”pair-correlation” approximation [1, 6]. Here (ij) denotes that two-pair combination of environment spins forms a distinct subspace, allows to significantly reduce the many-body complexity of the original Hamiltonian. Then any operators $\widehat{O}$ acting on different subspaces are completely independent, i.e. $[\widehat{O}_{(ij)}, \widehat{O}_{(mn)}] = 0$ if $(ij) \neq (mn)$.

Plugging Eq. (S5) in Eq. (S4) and reordering terms we obtain the two-time environment noise correlation function as

$$\langle \delta B(t)\delta B(0)\rangle \approx \sum_{i<j} \left\langle \delta B_{ij}(t)\delta B_{ij}(0)\right\rangle_{(ij)}, \text{ with } \delta B_{ij} = b_i I_i^z + b_j I_j^z. \tag{S6}$$

Here it offers a unique chance that the expectation value can be evaluated in the subspace (ij).

In the following we employ a technique similar to the method of moments [7–9]. We focus on H_e as

$$H_e = \gamma_I B \sum_i I_i^z + 4\sum_{i<,j} d_{ij} I_i^z I_j^z - \sum_{i<,j} d_{ij}(I_i^+ I_j^- + I_i^- I_j^+)\cos(\omega t), \tag{S7}$$

where a frequency variable ω is introduced for convenience (eventually we will take the interested limit $\omega = 0$). The flip-flop rate for a pair (ij) is given by Fermi's golden rule

$$R_{ij}(\omega) = T_{ij}^{-1}(\omega) = 2\pi d_{ij}^2 \rho_{ij}(\omega), \tag{S8}$$

with the density of states $\rho_{ij}(\omega) = \sum_{a,b} \left|\left\langle a \left| F_{ij} \right| b\right\rangle\right|^2 \delta(E_a - E_b - \omega)$. We label the two eigenstates of subspace (ij) by $|a\rangle$ and $|b\rangle$ and $F_{ij} = I_i^+ I_j^- + I_i^- I_j^+$.

We define the n-th moment as [7–9]

$$\langle \omega^n \rangle = \frac{\int_0^\infty \omega^n \rho_{ij}(\omega)}{\int_0^\infty \rho_{ij}(\omega)}. \tag{S9}$$

Accordingly, the zeroth moment and the second moment are given by the invariance of the trace

$$\begin{aligned} \langle \omega \rangle = \overline{\omega} &= 2C\mathrm{Tr}\{[H_e, F_{ij}] I_i^+ I_j^-\}, \\ \left\langle \omega^2 \right\rangle &= -C\mathrm{Tr}\{[H_e, F_{ij}]^2\}, \end{aligned} \tag{S10}$$

where C is a normalization constant. It is lengthy but straightforward to derive the following relations:

$$\langle \omega \rangle = \overline{\omega} = \frac{1}{2}\left|b_i - b_j\right| = \Delta_{ij}, \tag{S11}$$

$$\left\langle (\omega - \overline{\omega})^2 \right\rangle = 4\sum_{k\neq i,j} (d_{ik} - d_{jk})^2 = \sigma_{ij}^2. \tag{S12}$$

where we denote Δ_{ij} the frequency shift felt by the central ion when one flip-flop event takes place, and σ_{ij}^2 the linewidth for the flip-flop. Using these expressions we can discuss the shape of $\rho_{ij}(\omega)$, which leads to

$$\rho_{ij}(\omega) = \frac{1}{8\sqrt{2\pi}\sigma_{ij}}\left(\exp[-\frac{(\omega-\overline{\omega})^2}{2\sigma_{ij}^2}] + \exp[-\frac{(\omega+\overline{\omega})^2}{2\sigma_{ij}^2}]\right), \text{ with } \sigma_{ij} = \sqrt{\langle(\omega-\overline{\omega})^2\rangle}. \tag{S13}$$

The flip-flop rate is then obtained by setting $\omega = 0$ in Eq. (S13) and (S8),

$$R_{ij} = T_{ij}^{-1} = \frac{\sqrt{2\pi}}{4}\frac{d_{ij}^2}{\sigma_{ij}}\exp(-\frac{\Delta_{ij}^2}{2\sigma_{ij}^2}). \tag{S14}$$

i.e., Eq. (5) in the main text. Therefore we can calculate the correlation function

$$\langle \delta B(t)\delta B(0)\rangle = \frac{1}{4}\sum_i b_i^2 \sum_{i<j} P_{ij}\exp(-\frac{2t}{T_{ij}}), \text{ with } P_{ij} = \frac{R_{ij}\Delta_{ij}^2}{\sum_{n<m} R_{nm}\Delta_{nm}^2} \tag{S15}$$

Eq. (S15) allows realistic calculations of environment noise spectrum without using any phenomenological assumption. All we need are the rare-earth-doped crystal lattice parameters and the magnetic moments γ_s, γ_I available from the experimental literature [10], which in turn determine the fluctuation rate R_{ij} and the corresponding correlation function. For the studied system europium-ion dopant in yttrium orthosilicate (Eu^{3+}:Y_2SiO_5), Y_2SiO_5 have $C2/c(C_{2h}^6)$ space group with cell parameters $a = 1.041$ nm, $b = 0.673$ nm, $c = 1.249$ nm, $\beta = 103.65°$. Each cell contains 64 atoms and Si presents an unique crystallographic site, Y enters two different site in the lattice and Eu ion occupies site 1 of Y position. The Y's gyromagnetic moment is 2.09 MHz/T, while Eu ion's state has a large magnetic moment (be of the order of 100 MHz/T). The computation resources of calculations are in general rapidly increasing due to a large number of environment spins. In practice, we wrote a computer program that sums over lattice sites with 10^4 atoms and the numbers are high enough to guarantee convergence. The correlation function has been numerically evaluated in Fig. 1(b) in the main text and subsequently fitted to a exponential form $b^2 \exp(-t/\tau_c)$ in order to obtain the environment noise amplitude b and correlation time τ_c.

NUMERICAL SIMULATIONS OF COHERENCE BEHAVIORS

The calculation of environment spectrum outlined above provides good description for the bath around the central ion. The environment noise effects can be modelled [11],

$$H_{eff} = \epsilon_0 S_z + \epsilon_1 \delta B S_z + \epsilon_2 (\delta B)^2 S_z + ... \tag{S16}$$

where we expand the Hamiltonian in the perturbation of the fluctuating magnetic field δB: $\epsilon_1 = \frac{\partial \epsilon_0}{\partial B}$ and $\epsilon_2 = \frac{1}{2}\frac{\partial^2 \epsilon_0}{\partial B^2}$ with the correlation function $C(t) = \langle \delta\epsilon(t)\delta\epsilon(0)\rangle = b^2 \exp(-t/\tau_c)$.

In practice, we can simulate the evolution of the central ion Eq. (S16) by numerically solving the time-dependent Schrodinger equation. The numerical calculations utilize the Runger-Kutta scheme and the result is averaged over 500 realizations of the $\delta\epsilon$ noise of the environment. Each run has a stochastic term $\delta\epsilon$, and the initial value $\delta\epsilon(0)$ is sampled from the Gaussian distribution of the noise with width b. At each time step, the value of the random field $\delta\epsilon(t)$ can be randomly sampled using the previous value and the transition probability for the environment process with the correlation function $C(t)$. The simulation results for a wide range of dynamical decoupling protocols are presented in Figs 2, 3, 4 of the main text. Similar numerical analysis has been used in a variety of superconducting, quantum dot and NV center systems [11–13].

ANALYTICAL EXPRESSIONS OF COHERENCE BEHAVIORS

We quantify the coherence of the central ion by using the function $\exp[-\chi(t)]$, which is defined as the off-diagonal elements of the density matrix $|\langle\rho_{+-}(t)\rangle|$. Formally, the dynamics of the central ion can be analysed using the evolution operator [11]. Dynamical decoupling sequences can be considered as applying a certain number of π pulses at time t_i. Since the π pulse is given by $\exp(-\frac{i\pi\sigma_x}{2}) = -i\sigma_x$, we can write the evolution operator by slicing the time interval into small pieces at $t_1, t_2, ..t_n$,

$$U(t) = ...(-i\sigma_x)\exp\left(-\frac{i\sigma_z}{2}\int_{t_{i-1}}^{t_i}[\epsilon_0 + \delta\epsilon]dt\right)...(-i\sigma_x)\exp\left(-\frac{i\sigma_z}{2}\int_0^{t_1}[\epsilon_0 + \delta\epsilon]dt\right). \tag{S17}$$

When the fluctuations are Gaussian, the average over noise realizations can be expressed as a Gaussian functional integral over all possible values of $\delta\epsilon$. Therefore $\chi(t)$ can be calculated through the spectral density of the environment noise $S(\omega)$ as [11]

$$\chi(t) = \int_0^\infty \frac{d\omega}{\pi} S(\omega)\frac{F(\omega t)}{\omega^2}. \tag{S18}$$

Here $F(\omega t)$ is the filter function derived from the Fourier transform of the evolution of the pulse sequence on the ion,

$$F(\omega t) = \frac{1}{2}\left|\sum_{i=0}^{n}(-1)^i(e^{i\omega t_{i+1}} - e^{i\omega t_i})\right|^2. \tag{S19}$$

Analytical expressions of $F(\omega t)$ for the CPMG sequences under consideration can be obtained:

$$\begin{aligned} F(\omega t) &= 8\sin^4(\tfrac{\omega t}{4n})\sin^2(\tfrac{\omega t}{2})/\cos^2(\tfrac{\omega t}{2n}) \text{ for even } n, \\ F(\omega t) &= 8\sin^4(\tfrac{\omega t}{4n})\cos^2(\tfrac{\omega t}{2})/\cos^2(\tfrac{\omega t}{2n}) \text{ for odd } n. \end{aligned} \tag{S20}$$

We will consider the case for large n limit or pulse interval $\tau \ll \tau_c$ (i.e., the case of long correlation time τ_c which our system belongs to). For CPMG sequence $F(\omega t) < (\omega t/2n)^4$ for $\omega t < 2n$, i.e. $F(\omega t)$ strongly suppresses the low-frequency noise. Since the spectral density is a Lorentzian type $S(\omega) = \frac{2b^2/\tau_c}{\omega^2+1/\tau_c^2}$, the filter function $F(\omega t)$ will suppress the low-frequency part ($\omega < 1/\tau_c$). While for large ωt, the filter function can be approximated by a periodic train (with period $1/2\pi n$) of square peaks of height $2n^2$ and width $1/2n^2$. Thus the tail part as $\frac{2b^2/\tau_c}{\omega^2}(\omega > 1/\tau_c)$ contributes to the integral Eq. (S16). Then, to the lowest order of $1/n$, we can sum all of the terms of narrow peaks for $F(\omega t)$ and get

$$\chi(t) \approx \frac{(b\tau_c)^2}{12n^2}\left(\frac{t}{\tau_c}\right)^3. \tag{S21}$$

which is the result Eq. (11) in the main text.

ANALYSIS FOR A PRASEODYMIUM DOPED CRYSTAL

Several ion-doped crystals offer interesting perspectives as quantum memories for optical states. Among them, the best known systems with the required properties are $Eu^{3+}:Y_2SiO_5$ and $Pr^{3+}:Y_2SiO_5$ [14]. In the main text we have described the general theory of rare-earth-doped crystals and given the numerical results for a particular case ($Eu^{3+}:Y_2SiO_5$). This general theory can be directly applied to other ion-doped solids.

We start by calculating the correlation function of Y-bath spins in a $Pr^{3+}:Y_2SiO_5$ system. Fig. S1(a) shows that this correlation function decays smoothly over 1 second, and its behavior is fitted by an exponential form $b^2\exp(-t/\tau_c)$ with $b = 90$ Hz and $\tau_c = 0.5$ s. Then using this bath spin dynamics, Fig. S1(b) presents the signals of central Pr ion under dynamical decoupling pulses as a function of time. The coherence envelope can be compared with experimental result [15].For example, the calculated value of deocherence rate $1/T_2$ for pulse interval $\tau = 10$ ms is 6.8×10^{-2} s^{-1}, which is agreement with the measured gradient of the dynamical decoupling decay curve (4.7×10^{-2} s^{-1}).

Next we discuss its implications for the different systems. Clearly the results of $Pr^{3+}:Y_2SiO_5$ are quite similar to those of $Eu^{3+}:Y_2SiO_5$, but the time scale of coherence envelope is twenty times smaller than the value of Eu-doped system. Actually our theory suggests this small time scale is coming from the incomplete suppression of Y bath spin dynamics. The key mechanism is that the strong couplings between the central ion and the neighbour Y spins is responsible for that Larmor frequencies of neighbour Y spins are detuned from each other. Including the relatively smaller magnetic moment of Pr ion, the calculation shows that the couplings between the central ion and the neighbour Y spins is one order of magnitude smaller in $Pr^{3+}:Y_2SiO_5$ than those in $Eu^{3+}:Y_2SiO_5$. The smaller b_i reduces the degree of detuning of neighbour Y spins, hence increase the flip-flop rate R_{ij}. Therefore the Y bath spin dynamics is not entirely suppressed in $Pr^{3+}:Y_2SiO_5$ and the correlation time τ_c is only 0.5 s (in comparison, the correlation time is about 12 s in Eu-doped system). Furthermore, the scaling behaviors of coherence envelope extend over a range on several tens of seconds time scale (in comparison, the scaling spans an hour-long time scale in Eu-doped system).

[1] Yang, W., Ma, W.-L., & Liu, R.-B. Quantum many-body theory for electron spin decoherence in nanoscale nuclear spin baths. *Rep. Prog. Phys.* **80**, 016001 (2017).

[2] Dobrovitski, V. V., Feiguin, A. E., Hanson, R., & Awschalom, D. D. Decay of rabi oscillations by dipolar-coupled dynamical spin environments. *Phys. Rev. Lett.* **102**, 237601 (2009).

[3] de Lange, G., Wang, Z. H., Riste, D., Dobrovitski, V. V., & Hanson, R. Universal dynamical decoupling of a single solid-

state spin from a spin bath. *Science* **330**, 60-63 (2010).

[4] Witzel W. M. & Das Sarma, S. Quantum theory for electron spin decoherence induced by nuclear spin dynamics in semiconductor quantum computer architectures: Spectral diffusion of localized electron spins in the nuclear solid-state environment. *Phys. Rev. B* **74,** 035322 (2006).

[5] de Sousa, R. Electron Spin as a spectrometer of nuclear-spin noise and other fluctuations. *Top. Appl. Phys.* **115,** 183 (2009).

[6] Yao, W., Liu, R.-B., & Sham, L. J. Theory of electron spin decoherence by interacting nuclear spins in a quantum dot. *Phys. Rev. B* **74**, 195301 (2006).

[7] Van Vleck, J. H. The dipolar broadening of magnetic resonance lines in crystals. *Phys. Rev.* **74**, 1168-1183 (1948).

[8] Abragam, A. *The Principles of Nuclear Magnetism* (Oxford University Press, London, 1961).

[9] Slichter, C. P. *Principles of Magnetic Resonance* (Springer-Verlag, Berlin, 1996).

[10] Zhong, M., Hedges, M. P., Ahlefeldt, R. L., Bartholomew, J. G., Beavan, S. E., Wittig, S. M., Longdell J. J., & Sellars, M. J. Optically addressable nuclear spins in a solid with a six-hour coherence time. *Nature* **517**, 177-180 (2015).

[11] Bylander, J., Gustavsson, S., Yan, F., Yoshihara, F., Harrabi, K., Fitch, G., Cory, D. G., Nakamura, Y., Tsai, J.-S., & Oliver, W. D. Noise spectroscopy through dynamical decoupling with a superconducting flux qubit. *Nature Physics* **7**, 565-570 (2011).

[12] Shi, Z., Simmons, C. B., Ward, D. R., Prance, J. R., Wu, X., Koh, T. S., Gamble, J. K., Savage, D. E., Lagally, M. G., Friesen, M., Coppersmith S. N., & Eriksson, M. A. Fast coherent manipulation of three-electron states in a double quantum dot. *Nature Communications* **5**, 3020 (2104).

[13] van der Sar, T., Wang, Z. H., Blok, M. S., Bernien, H., Taminiau, T. H., Toyli, D. M., Lidar, D. A., Awschalom, D. D., Hanson R., & Dobrovitski, V. V. Decoherence-protected quantum gates for a hybrid solid-state spin register. *Natur*e **484**, 82-86 (2012).

[14] de Riedmatten, H., & Afzelius, M. *Engineering the Atom-Photon Interaction*, edited by Predojevic, A. & Mitchell, M. W. (Springer, 2015).

[15] Fraval, E., Sellars, M. J., & Longdell, J. J. Dynamic decoherence control of a solid-state nuclear-quadrupole qubit. *Phys. Rev. Lett.* **95**, 030506 (2005).

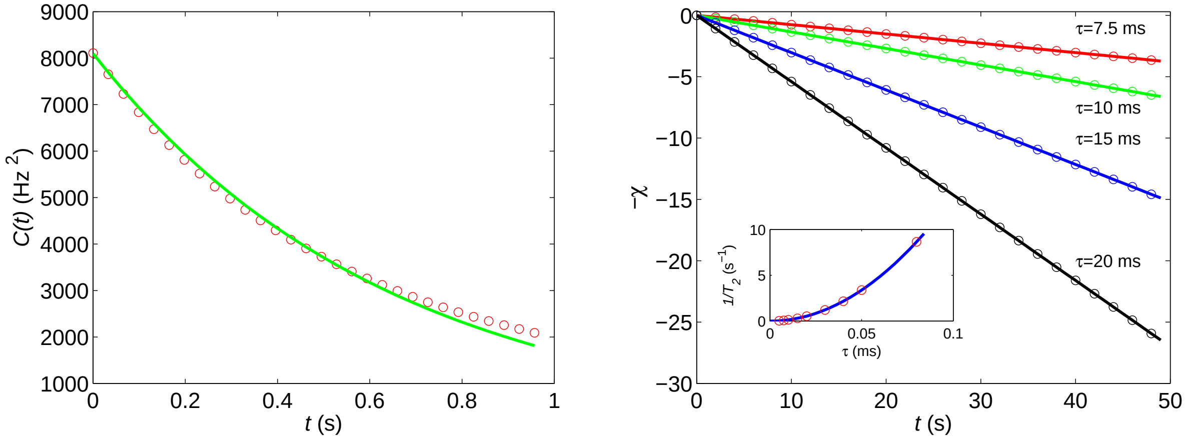

(a) (b)

FIG. 1. (a) Numerically calculated environment correlation function $C(t)$ for Pr-doped crystals (red circles) and the fitted shape $b^2 \exp(-t/\tau_c)$ (green solid line) with $b = 90$ Hz and correlation time $\tau_c = 0.5$ s. (b) Circles are simulation data of coherence envelope for Pr-doped crystals under CPMG sequences with pulse interval $\tau = 7.5$ ms, 10 ms, 15 ms, 20 ms. Solid lines are obtained by the scaling expression $(t/T_2)^\beta$ with $\beta = 1$. Insert: Extracted $1/T_2$ for CPMG pulse interval. Solid line is also the scaling function $1/T_2 \sim \tau^\delta$ with $\delta = 2$.
